# Management and outcome of adult generalized tetanus in a Chinese tertiary hospital

**DOI:** 10.3389/fpubh.2024.1301724

**Published:** 2024-02-15

**Authors:** Yuling An, Yi Guo, Lijuan Li, Ziyu Li, Mingming Fan, You Peng, Xiaomeng Yi, Haijin Lv

**Affiliations:** ^1^Department of Surgical Intensive Care Unit, Third Affiliated Hospital of Sun Yat-sen University, Guangzhou, China; ^2^Yunnan Fuwai Cardiovascular Hospital, Kunming, China; ^3^Third Affiliated Hospital of Sun Yat-sen University, Guangzhou, China

**Keywords:** tetanus, surgical infectious disease, prognosis, autonomic instability, ICU

## Abstract

**Background:**

Tetanus is a rare surgical infectious disease with a high reported relevant mortality. It still remains a serious problem in public health, particularly in low-income and middle-income countries. The purpose of this study was to investigate the management and prognosis of adult generalized tetanus in our hospital.

**Methods:**

A total of 20 adult generalized tetanus patients were recruited in this retrospective observational study. Patients were retrieved from the hospital data base via discharge diagnosis. Patients were divided into two groups (Severe or Non-severe tetanus group) based on the severity of tetanus by using the Ablett classification. The differences between the two groups were compared.

**Results:**

The study included 11 males (55%) and 9 females (45%). All tetanus patients recovered. The median age was 53.5 years [IQR: 19–78]. There were 1 mild (Grade 1) case (5%),5 moderate (Grade 2) cases (25%), 2 severe (Grade 3) cases (10%), and 12 very severe (Grade 4) cases (60%). Nineteen patients (95%) did not have tetanus immunization before. The majority of patients were farmers (60%), and came from rural areas (60%). Thirteen (65%) patients had a history of puncture injury. The rate of wound debridement after admission was 60% overall. Thirteen (65%) patients required mechanical ventilation for a median of 21 [IQR:12–41] days. Autonomic instability occurred in 13 (65%) patients. Pulmonary infections occurred in 12 (60%) patients. Median duration of hospital stay was 29.5 [IQR:12–68] days. More patients in the Severe group needed ICU admission, wound debridement, mechanical ventilation and heavy sedation combined with muscle relaxants (*p* < 0.05). The hospital stay was significantly longer in patients in the Severe group (*p* < 0.05).

**Conclusion:**

After effective treatment, all adult patients with generalized tetanus in this study were cured and discharged. Severe tetanus requires early ICU treatment, wound debridement and effective treatment of autonomic instability.

## 1 Introduction

Tetanus is an acute, often fatal disease caused by tetanus toxin, which is a neurotoxin produced by *Clostridium tetani* (1). Tetanus toxin can block the release of neurotransmitters that inhibit muscle contraction, resulting in spontaneous muscle spasm and overall body rigidity. Clinically, the disease is manifested with a lockjaw (trismus), a snarling smile (rhisus sardonicus), pain-related involuntary muscle contraction and involuntary urination and defecation (2).

Tetanus is often endemic, and the incidence rate increases after natural disasters (3). Over the past decade, extensive vaccination coverage for children has led to very low incidence rate of tetanus all over the world. The incidence of tetanus was about 1/10,000,000 in the US (4). Poland reported 17 cases of tetanus with a total incidence rate of 0.44/1,000,000 from 2018 to 2019 (5). Today the majority of new cases of tetanus occur in South Asia and Sub-Saharan Africa, which account for 82% of all tetanus cases globally. In 2019, India reported 7,071 cases of tetanus to the World Health Organization (WHO), accounting for about half of the world’s reported cases (6). However, some of immunization services have been affected by COVID-19, which resulted in 25 million children worldwide missing out on vaccination in 2021.

Similarly, according to the Global Burden of Disease (GBD) study, 77% of 38,000 deaths from tetanus occured in South Asia and Sub-Saharan Africa in 2017 (7). The mortality rate of adult tetanus in developing countries varies greatly, ranging from 10 to 50%. The lack of health resources and appropriately trained staff means that the mortality rate of tetanus is still high in some developing countries (8, 9), with an estimated 48.8% deaths in African countries (10). Due to incomplete mortality data for many countries, the actual number of tetanus-related deaths may be larger than current. Therefore, it is very important to study the factors related to the mortality of infected patients.

Therapy of tetanus involves providing supportive care, wound cleaning, antibiotic eradication of *C. tetani*, neutralizing circulating toxin and managing potential complications of severe muscle spasms, including acute respiratory failure, asphyxiation, pneumonia, rhabdomyolysis, pulmonary emboli and hemodynamic instability. In addition to the disease process, the high mortality may be due to poor diagnosing,wound care and lack of awareness regarding immunization in developing countries (6). The purpose of this study was to analyze the demographic, clinical and therapeutic factors and mortality of adult tetanus patients in our hospital. It was expected that the findings would provide clinicians with new data and may help improve the future management strategies for tetanus.

## 2 Materials and methods

### 2.1 Study design and patients

The study was approved by the Ethics Committee of third Affiliated Hospital of Sun Yat-Sen University for the analysis of the data from the database and conducted in accordance with the Helsinki Declaration of 1975. We reviewed the hospital database that involved adult tetanus patients who were admitted to the Third Affiliated Hospital of Sun Yat-Sen University (Guangzhou, China) between January 2012 and May 2022. The diagnosis of tetanus conformed to the WHO definition of tetanus. The study obtained the informed consent of all patients.

The inclusion criteria included patients over 18 years old and with a discharge diagnosis of tetanus during the period of the study. The diagnosis of tetanus was entirely clinical and based on the history of the wound and the presence of one or more of the following: (i) Lockjaw or snarling smile, (ii) Muscle spasm, (iii) Rigidity of the neck and/or abdomen/neck stiffness. Data analysis was based on hospital records. The following data were collected: age, sex, occupation, type of injury, culture of wound tissue or tissue secretions,incubation period, comorbidity, antibiotics, whether to use mechanical ventilation, the acute physiology and chronic health evaluation II (APACHE II) score at admission, tetanus antitoxin or tetanus immunoglobulin dose, sedatives and analgesics, types of muscle relaxant used, length of stay in ICU, hospital duration, autonomic nervous disorders and pneumonia (determined by chest).

Severity of tetanus was classified as the Ablett grading system: Grade 1(mild), mild to moderate trismus with no respiratory compromise; Grade 2 (moderate), moderate trismus and moderate respiratory compromise with an increased respiratory rate (>30 breaths per min); Grade 3(severe), severe trismus with increased respiratory rate (>40 breaths per min) and tachycardia (>120 beats per min); Grade 4 (very severe), severe manifestations with autonomic dysfunction (11). We divided all those patients into two group, including Severe or Non-severe tetanus group. The Severe group involved the very severe (grade 4) and severe (grade 3) cases, while the Non-severe group involved the moderate (grade 2) and mild (grade 1) cases.

### 2.2 Statistical analyses

SPSS 25.0 statistical software (IBM Corporation, Chicago, IL, United States) was used for data analysis. Fisher’s exact probability method was used to compare the rates of the two groups. The median (IQR) was used for the Non-normally distributed data, and the rank sum test was used to compare the two groups. A *p* value <0.05 was considered to be statistically significant.

## 3 Results

### 3.1 Demographic data and clinical features

Twenty patients were admitted to our hospital from January 1, 2012 through May 1, 2022. Demographic data and clinical features were shown in Tables 1–3. Eleven males and 9 females had been involved in this study and the median age was 53.5 years (IQR: 19–78). The average acute physiology and chronic health evaluationII (APACHE II) score at admission was 13.5(IQR: 11–18). All the clinical classification of tetanus was generalized. Except for a 19 years old young man who had received diphtheria-pertussis-tetanus (DPT) immunization, the rest of patients did not know their previous history of tetanus immunization. The majority of patients were farmers (12 cases, 60%), and came from rural areas (12 cases, 60%). The incubation period was not available for one patient with unknown routes of infection. The remaining 19 patients had an average incubation period of 8 days (IQR: 3–15). Acute injury of extremities was the main entrance of *Clostridium tetani* infection. Nineteen patients had a clear history of trauma. Puncture injury was the most common type of injury types. Only one patient had cultivated *Clostridium tetani* in wound tissue or tissue secretion. Three cases (15%) were diagnosed incorrectly by the initial physician and others. Total 13 cases (65%) had autonomic disorder and 8 cases (40%) had at least one comorbidity.

**Table 1:** Details of tetanus patients (*n* = 20).

Patient	Age, (years)	Sex	History of trauma	Site of injury	Debridement (Yes/No)	Autonomic disorder (yes/no)	Intubation time (days)	incubation period, (days)	Length of stay (LOS)	Severity score	APACHE-II at day 0
1	52	M	Stab wound	toe	No	No	0	12	31	Grade 2	13
2	19	M	Knife cut	hand	No	No	0	8	15	Grade 2	11
3	57	F	Stab wound	foot	Yes	Yes	0	15	20	Grade 2	14
4	55	F	unknown	unknown	No	No	0	Unknown	12	Grade 1	15
5	47	F	Knife cut	leg	No	No	0	13	27	Grade 2	13
6	35	M	Stone scratch	leg	No	No	0	3	12	Grade 2	12
7	45	M	Stab wound	foot	No	No	0	7	20	Grade 3	13
8	51	F	Stab wound	toe	Yes	Yes	21	6	53	Grade 4	13
9	45	M	Stab wound	foot	Yes	Yes	12	5	26	Grade 4	13
10	56	M	Smash injury	arm	Yes	Yes	28	10	51	Grade 4	14
11	56	F	Shovel cuts	leg	Yes	Yes	15	10	38	Grade 4	14
12	58	M	Stab wound	toe	Yes	Yes	10	6	25	Grade 4	15
13	62	M	Stab wound	toe	Yes	Yes	14	10	26	Grade 4	15
14	72	F	Stab wound	finger	Yes	Yes	20	12	29	Grade 4	17
15	42	F	Stab wound	foot	Yes	Yes	30	10	43	Grade 4	12
16	78	M	Stone scratch	leg	No	Yes	20	8	35	Grade 3	18
17	62	M	Stab wound	finger	Yes	Yes	23	10	52	Grade 4	16
18	43	F	Stab wound	foot	Yes	Yes	41	6	68	Grade 4	13
19	57	F	Stab wound	finger	Yes	Yes	23	7	30	Grade 4	14
20	42	M	Stab wound	toe	Yes	Yes	23	7	31	Grade 4	13

**Table 2:** Demographic data of the patients (*n* = 20).

Variable	Total *n* (%)	Severe group *n* (%)	Non-severe group *n* (%)	*p*-value
Age (years), median (IQR†)	53.5 (19–78)	56 (42–78)	49.5 (19–57)	0.19
Age category (years)				0.08
< 40	2 (10%)	0	2 (33.3%)	
≥40	18 (90%)	14 (70%)	4 (66.7%)	
Gender				1.00
Male	11 (55%)	8 (57.1%)	3 (50%)	
Female	9 (45%)	6 (42.9%)	3 (50%)	
Occupation				0.01
Farmer	12 (60%)	11 (78.6%)	1 (16.7%)	
Labor worker	3 (15%)	2 (14.3%)	1 (16.7%)	
Other occupation	5 (25%)	1 (7.1%)	4 (66.7%)	
Residence				0.02
Rural	12 (60%)	11 (78.6%)	1 (16.7%)	
Urban	8 (40%)	3 (21.4%)	5 (83.3)	

**Table 3:** Clinical characteristics of the patients (*n* = 20).

Variable	Total *n* (%)	Severe group *n* (%)	Non-severe group *n* (%)	*P*-value
Immunization history				
Yes	1 (5%)	0	1 (16.7%)	0.30
No	19 (95)	14 (100%)	5 (83.3%)	
Postexposure prophylaxis				1.0
Yes	1 (5%)	1 (7.1%)	0	
No	19 (95)	13 (92.9%)	6 (100%)	
Wound type				0.30
Puncture	13 (65%)	11 (78.6%)	3 (50%)	
Other injury type	6 (30%)	3 (21.4%)	3 (50%)	
Location of wound				0.27
Upper limbs	5 (25%)	4 (28.6%)	1 (16.7)	
Lower limbs	14 (70%)	10 (71.4%)	4 (66.7%)	
Not identified	1 (5%)	0	1 (16.7%)	
Wound cleaning before admission				1.0
Yes	3 (15%)	2 (14.3%)	1 (16.7%)	
No	17 (85%)	12 (85.7%)	5 (83.3%)	
First clinical symptoms				
Lockjaw	16 (80%)	14 (100%)	2 (33.3%)	0.80
Snarling smile	17 (85%)	14 (100%)	3 (50%)	
Muscle spasm	11 (55%)	10 (71.4%)	1 (16.7%)	
First diagnosis correctly				
Yes	17 (85%)	13 (92.9%)	4 (66.7%)	0.20
No	3 (15%)	1 (7.1%)	2 (33.3%)	
Autonomic instability				0.0002
Yes	13 (65%)	13 (92.9%)	0	
No	7 (35%)	1 (7.1%)	6 (100%)	
Comorbidity.				0.77
Hypertension	3 (15%)	3 (%)	1 (%)	
Diabetes mellitus,	2 (10%)	2 (%)	0	
COPD	1 (5%)	1 (%)	0	
Ischemic heart disease	1 (5%)	1 (%)	0	
Onset time (days)				0.16
< 2	11 (55%)	10 (71.4%)	2 (33.3%)	
≥2	9 (45%)	4 (28.6%)	4 (66.7%)	
Incubation period (days), median (IQR)	8 (3–15)	7.5 (5–12)	12 (3–15)	0.21
APACHE-II at day 0, median (IQR)	13.5 (11–18).	14 (12–18)	13 (11–15)	0.16

Comparing the Severe group (14 cases) and the Non-severe group (6 cases), there were no significant differences between the two groups in age, gender, incubation time, wound type, APACHE II score at admission and type of antibiotic used. Compared with the Non-severe group, more severe patients were farmers lived in rural areas (*p* < 0.05). The incidence of autonomic instability symptoms in the Severe group were higher than those in the Non-severe group (*p* < 0.05).

### 3.2 Therapeutic parameters and outcome

All the patients were isolated as far as possible after diagnosis to avoid all kinds of stimulation. Metronidazole was the most commonly used antibiotic (18 cases, 90%) followed by Penicillin (12 cases, 60%). Either tetanus antitoxin (TAT) or tetanus immunoglobulin (TIG) was used to neutralize the toxin in early stage. After skin test or desensitization, TAT was administered intravenously for 7–10 days (30–50 thousand U/D). Human tetanus immunoglobulin (TIG)3,000 ~ 6,000 U, multi-point one-time deep intramuscular injection. Sedative drugs and muscle relaxation drugs were used to treat spasticity and autonomic symptoms. Richmond Agitation Sedation Scale (RASS) was used to assess the degree of sedation (12). The sedation goals were to maintain moderate to deep sedation (i.e., RASS maintained at −3 to −4 points) during the onset of typical symptoms and then to maintain mild sedation (i.e., RASS maintained at −1 to – 2 points) as symptoms improved and vital signs did not fluctuate significantly. Thirteen patients (65%) were treated with tracheal intubation or tracheotomy and follow-up supportive treatment with invasive mechanical ventilator. The median mechanical ventilation period was 21 days (IQR10-41). The rate of wound debridement after admission was 60% overall. Pulmonary infections occurred in 12 (60%) of patients. The median duration of hospital stay was 29.5 days (IQR: 12–68 days) (Table 4).

**Table 4:** Therapeutic parameters and outcome of the patients (*n* = 20).

Variable	Total *n* (%)	Severe group *n* (%)	Non-severe group *n* (%)	*P*-value
Type of antibiotic used				0.07
Metronidazole	10 (50%)	8 (57.1%)	2 (33.3%)	
Combined Metronidazole and Penicillin	8 (40%)	6 (42.9%)	2 (33.3%)	
Other antibiotic	2 (10%)	0	2 (33.3%)	
ICU admission (Yes/No)				0.0004
Yes	15 (75%)	14 (100%)	1 (16.7%)	
No	5 (25%)	0	5 (83.3%)	
Debridement after admission				0.0007
Yes	12 (60%)	12 (85.7%)	0	
No	8 (40%)	2 (14.3%)	6 (100%)	
Invasive mechanical ventilation				0.0002
Yes	13 (65%)	13 (92.9%)	0	
No	7 (35%)	1 (7.1%)	6 (100%)	
Heavy sedation combined with muscle relaxants				0.0007
Yes	12 (60%)	12 (85.7%)	0	
No	8 (40%)	2 (14.3%)	6 (100%)	
Feeding after pylorus				0.12
Yes	6 (30%)	6 (42.9%)	0	
No	14 (70%)	8 (57.1%)	6 (100%)	
Pulmonary infection				0.16
Yes	12 (60%)	10 (71.4%)	2 (33.3%)	
No	8 (40%)	4 (28.6%)	4 (66.7%)	
Sequela				1.0
Yes	2 (10%)	2 (14.3%)	0	
No	18 (90%)	12 (85.7%)	6 (100%)	
Hospital stay (d) median (IQR†)	29.5 (12–68)	33 (21–68)	17.5 (12–31)	0.008

All the tetanus patients in our study were successfully cured and discharged and had a follow-up at least 3 months. Except for two patients with mild limb stiffness, all the other patients recovered well without any sequela (Table 4).

Comparing the Severe group and the Non-severe group, there were no significant differences between the two groups in incidence of pulmonary infection, type of antibiotic used and feeding mode. More patients in the Severe group needed ICU admission, wound debridement, mechanical ventilation and heavy sedation combined with muscle relaxants (*p* < 0.05). The hospital stay was significantly longer in patients in the Severe group (*p* < 0.05) (Table 4).

## 4 Discussion

Tetanus is a rare infectious disease in the western world. The main reason is that tetanus toxoid is widely vaccinated from infants to adults. However, in developing countries, it is still prevalent due to lack of immunity or failure to vaccinate with booster within 10 years after the previous series of vaccination (6). We described the clinical characteristics and results of a large cohort study of adult generalized tetanus patients who were treated in a tertiary hospital in China. The mortality rate in this study was zero. To our knowledge, this is the lowest reported mortality rate for a series of tetanus patients worldwide, in stark contrast to some low-income and middle-income countries where, despite mechanical ventilation, the mortality rate was as high as 44.5 to 57.8% (13, 14).

The most effective preventive measure for tetanus is tetanus vaccination. In China, the government has offered diphtheria–pertussis–tetanus vaccines to children since 1978. In 2012, China was confirmed to have eliminated maternal and neonatal tetanus (15). However, like many other low-income and middle-income countries, there are no guidelines with respect to tetanus immunization for adults in China (16). Recently a cross-sectional analysis of serum antibody titers in 546 adult subjects from United States demonstrated that durable levels of protective antitoxin immunity existed in the majority of tetanus vaccinated individuals, 95% of the population could last for more than 30 years. This suggested that it may no longer be necessary to administer booster vaccinations every 10 years (17). Nineteen patients (95%) were not vaccinated in our study, farmers accounted for the majority of tetanus patients, which was similar to other studies in China and Bangladesh (9, 18). Farmers in low-income and middle-income countries often work barefoot and are more exposed without adequate personal protection (19). Therefore, they can be considered as high-risk groups and give priority to primary and enhanced immunization as appropriate. In around 20% of cases, no identifiable wound is present (3). Therefore, misdiagnosis of tetanus often occured, especially when the wound was slight or even forgotten (20). A report from a hospital in Japan showed only 4 out of 11 cases were diagnosed correctly by the initial physician and others (21). In our study, 3 patients were first misdiagnosed as chest pain, stroke and epileps. We advocated screening high-risk groups among adults, the vulnerable adult population should be identified and vaccinated to protect them from tetanus. It can even be considered as an effective alternative or revision to the World Health Organization’s policy on appropriate vaccination for adults.

We believe that the good results of this study are mainly attributed to a professional team with extensive experience in tetanus treatment and care. Tetanus patients should be managed in a hospital with good intensive care facilities, including invasive hemodynamic monitoring, mechanical ventilation and good infection control measures. When modern supportive treatment was available, most tetanus patients had reasonable cure and low death rates (17, 18). Death was usually caused by hospital infection or severe autonomic instability with arrhythmia (17). Circulatory disturbances due to autonomic instability is now one of the most challenging factors in the treatment of severe tetanus (22, 23). Autonomic instability usually peaks in the second week and subsides after the third week. This period is extremely unstable, characterized by malignant hypertension, tachycardia, hypotension or bradycardia (6). Autonomic instability can be treated with sedatives, anesthetics, clonidine, dexmedetomidine and b-blockers (24–27). In our study, the wound debridement rate of severe tetanus and the incidence of autonomic instability were both high, so the measures affecting survival may be active wound debridement and treatment of autonomic instability.

Whenever a wound occurs, proper wound care and vaccination can prevent tetanus (28). To prevent the generation of toxins, early and appropriate debridement of infected wounds, including concealed wound debridement, removal of foreign bodies and dead tissues, and abscess drainage, may be more effective than the use of antibiotics alone (11). However, there is no uniform guideline for wound debridement in patients with tetanus. Some published studies on the prognosis of tetanus have not reported the rate of implementation of debridement (10, 29, 30). We believe that debridement is essential to control the symptoms of tetanus and improve the prognosis, especially for severe tetanus. We advocate that debridement should be carried out on the premise that the patient’s airway has been unblocked and there is enough analgesic medication.

Tetanus has a long course of disease, lasting for 4–6 weeks (22). For the 6 patients who had severe stomach retention in this study, we used the nasojejunal tube for postpyloric feeding to strengthen nutritional support. The overall median duration of hospitalization of patients with tetanus in this study was 29.5 days, which was similar to a recent report of low mortality (2.8%) from Vietnam (30). The median hospital stay of the latter was 25 days, which was much longer than the hospitals reported in some areas with high mortality (10, 18). Severe tetanus, in particular, requires longer ICU time, more mechanical ventilation requirements and longer hospital stay, remains a significant burden on healthcare services.

## 5 Limitations

This study also had some limitations. First, this was a retrospective study and the retrospective design may introduce some selection bias or recall bias. Secondly, this was a single center study with a relatively small sample size. Third, the city where our hospital located is one of the relatively developed cities in China, which can not represent the overall medical treatment level of China.

## 6 Conclusion

We reported management and outcome features in a large contemporary cohort of patients in a single center. The survival rate of these patients was relatively high compared to other reported case series. Severe tetanus patients needed early mechanical ventilation support and effective management of complications and comorbidities. However, other results, such as hospital stay and mechanical ventilation requirements, indicate that tetanus remains a significant burden on medical services.

## Data availability statement

The original contributions presented in the study are included in the article/supplementary material, further inquiries can be directed to the corresponding author/s.

## Ethics statement

The studies involving humans were approved by the Ethics Committee of third Affiliated Hospital of Sun Yat-sen University. The studies were conducted in accordance with the local legislation and institutional requirements. The participants provided their written informed consent to participate in this study.

## Author contributions

YA: Conceptualization, Supervision, Writing – original draft, Resources. YG: Data curation, Writing – original draft. LL: Data curation, Writing – review & editing. ZL: Formal analysis, Software, Writing – review & editing. MF: Investigation, Validation, Writing – review & editing. YP: Investigation, Software, Writing – review & editing. XY: Data curation, Supervision, Writing – review & editing. HL: Project administration, Supervision, Writing – review & editing.
